# Vasoinhibins May Contribute to Postpartum Depression

**DOI:** 10.3389/fpsyt.2017.00167

**Published:** 2017-09-12

**Authors:** Jakob Triebel, Gonzalo Martínez de la Escalera, Carmen Clapp, Thomas Bertsch

**Affiliations:** ^1^Laboratory Medicine and Transfusion Medicine, Institute for Clinical Chemistry, Nuremberg General Hospital, Paracelsus Medical University, Nuremberg, Germany; ^2^Instituto de Neurobiología, Universidad Nacional Autónoma de México (UNAM), Querétaro, México

**Keywords:** vasoinhibins, prolactin/vasoinhibin axis, postpartum depression, 16K prolactin, prolactin

The Diagnostic and Statistical Manual of Mental Disorders lists the peripartum period as an onset specifier for major depressive disorder, where “peripartum period” is defined as the time of the “most recent episode during pregnancy as well as 4 weeks following delivery” (1). In a recent article, Rosman et al. reported that patients suffering from peripartum cardiomyopathy (PPCM) demonstrated a particularly high prevalence of depression, compared to healthy females of similar age, postpartum women, and other heart failure patients (2). “Peripartum cardiomyopathy is an idiopathic cardiomyopathy presenting with heart failure secondary to left ventricular systolic dysfunction toward the end of pregnancy or in the month following delivery, where no other cause of heart failure is found” (3). PPCM is relatively rare. The incidence for PPCM was reported to be 1 per 968 live births in the United States (4), and 1 per 10,149 in Denmark, respectively (5). As mentioned, the disease is idiopathic, meaning that no specific causal mechanism is known. However, a seminal study in 2007 reported that a 16 kDa vasoinhibin isoform may be a causal factor in the development of PPCM (6). Vasoinhibins inhibit the proliferation, permeability, and dilation of blood vessels and are generated by the proteolytic cleavage of their precursor molecule prolactin (PRL), the pituitary hormone colloquially referred to as the “nursing hormone” (7, 8). PRL increases during pregnancy in preparation for lactation and remains elevated during the postpartum period in the nursing mothers (9). It has been suggested that in PPCM, the 16 kDa vasoinhibin isoform is excessively generated and exerts a detrimental effect on the microvascularization of the myocardium, resulting in left ventricular dysfunction. Elevated circulating vasoinhibin levels have been demonstrated in patients with PPCM (6). A clinical trial investigating bromocriptine effectiveness to suppress vasoinhibin generation in patients with PPCM is underway (10) (www.clinicaltrials.gov; identifier: NCT00998556).

Women with PPCM may have an inherently higher risk for depression due to the prognostic uncertainty associated with PPCM, as argued by Rosman et al. (2). Also, genetic and non-endocrine factors may contribute to the complex etiopathology of postpartum depression (11–13). However, due to the profound hormonal changes during pregnancy and postpartum, endocrine factors have been often investigated as additional contributors to postpartum depression etiopathology (14, 15). Interestingly, not only have vasoinhibins been reported to be causally linked to PPCM but also to induce anxiety- and depression-related behaviors (16). After the intraventricular administration of vasoinhibins, rats exhibit increased anxiety and depression when subjected to the open field test, the forced swim test, or the elevated plus-maze; and vasoinhibins have direct effects on neuronal function (17). Moreover, vasoinhibin plasma levels are elevated in normal human pregnancy (18) and appear further elevated in patients with PPCM (6). A likely consequence is a surge of vasoinhibins in the cerebrospinal fluid (CSF). This is because PRL is a normal constituent of CSF and CSF PRL levels are a function of plasma PRL levels (19, 20), and elevation of circulating PRL levels leads to the accumulation of vasoinhibins across the retinal blood barrier. Along this line, it can be speculated that elevated vasoinhibins in the CSF of peripartum women, and even more so in those with PPCM, may contribute to the development of postpartum depression.

The high prevalence of postpartum depression in patients with PPCM reported by Rosman et al. (2), the causative role of vasoinhibins in PPCM (6), the elevated circulating levels of vasoinhibins in patients with PPCM (6), and the anxiogenic and depressive properties of vasoinhibins (16) highlight the need for investigating the contribution of vasoinhibins to postpartum depression and the putative value of their circulating levels as risk factors for this condition.

## Author Contributions

JT wrote the manuscript. GE, CC, and TB edited and revised the manuscript. All authors approved the final version of the manuscript.

## Conflict of Interest Statement

The authors declare that the research was conducted in the absence of any commercial or financial relationships that could be construed as a potential conflict of interest.
